# CTAB as a soft template for modified clay as filler in active packaging

**DOI:** 10.1016/j.dib.2015.02.002

**Published:** 2015-02-14

**Authors:** Kajonpop Rittirong, Suvit Uasopon, Paveena Prachayawasin, Nukul Euaphantasate, Kamon Aiempanakit, Sarute Ummartyotin

**Affiliations:** a Department of Physics, Faculty of Science and Technology, Thammasat University, Klong Luang, Patumtani 12120, Thailand; b National Metal and Materials Technology Center, Klong Luang, Patumtani 12120, Thailand

**Keywords:** Modified clay, CTAB, Surfactant template, Active packaging

## Abstract

The role of modified clay has been employed in many areas of engineering research. Structure of clay was mainly focused on alumino-silicate layer and its form was presented as pillar layer. It composed of many ion exchanges inside. In industry, in order to use clay with higher efficiency, modification on surface and porosity has been developed. CTAB, one of the most effective cationic surfactant, was employed to modify the surface and porosity of clay.

**Table:** **Specificationstable**

Subject area	*Physics*

More specific subject area	*Materials physics and surface modification*
Type of data	*Figure*
How data was acquired	*X-ray diffraction pattern, scanning electron microscope*
Data format	*Analyzed*
Experimental factors	*Chemical modification on surface of clay by using CTAB as a soft template. Effect of temperature, reaction time and solvent were investigated on surfactant template of modification. Clay was successfully modified by surfactant template method*
Experimental features	*Chemical modification*
Data source location	*Department of Physics, Faculty of Science and Technology, Thammasat University, Thailand*
Data accessibility	*Short data is available in this data article*.

**Value of the data**

• The data was associated with conventional synthetic route for clay modification for food packaging.
• The data provided the insightful reaction on the use of CTAB as cationic surfactant for clay modification.
• The research is valuable for food packaging research on water absorption and gas permeation.
• Research data was interesting for plastic based composite.

## 1 Data, experimental design, materials and methods

### 1.1 Data

**Fig. 1:**
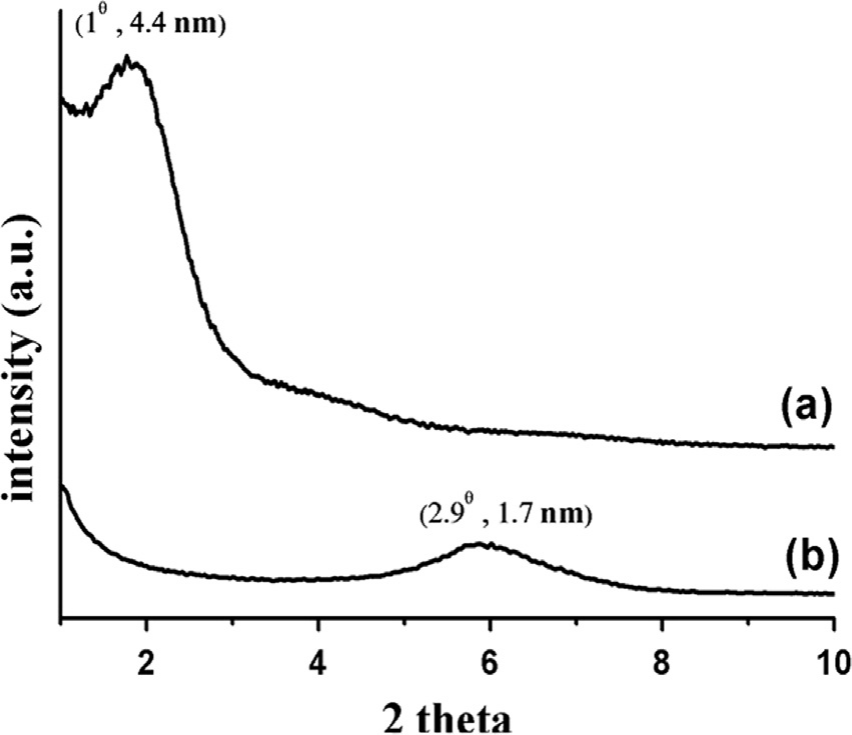
X-ray diffraction pattern of (a) modified clay and (b) bentonite.

Research data was involved on clay modification. Modified clay was successfully modified by conventional synthetic route [1]. It can be seen that the powder present the character of white-like powder. From the structural point of view, X-ray diffraction was employed to identify the crystal structure. The corresponding XRD pattern showed the presence of the diffraction peak. As seen from Fig. 1, the d-spacing of BTN was 1.7 nm (Fig. 1b). After chemical modification by using CTAB, strong evidence on 1° was observed. It can be calculated by using Bragg׳s law that the d-spacing of modified porous clay was to be 4.4 nm. The small angle region was observed and it was noted that structure of porous clay was significantly changed. The distance between layers was therefore extent.

This was probably due to the fact that the chemical modification process was involved on surfactant template modification [2].

**Fig. 2:**
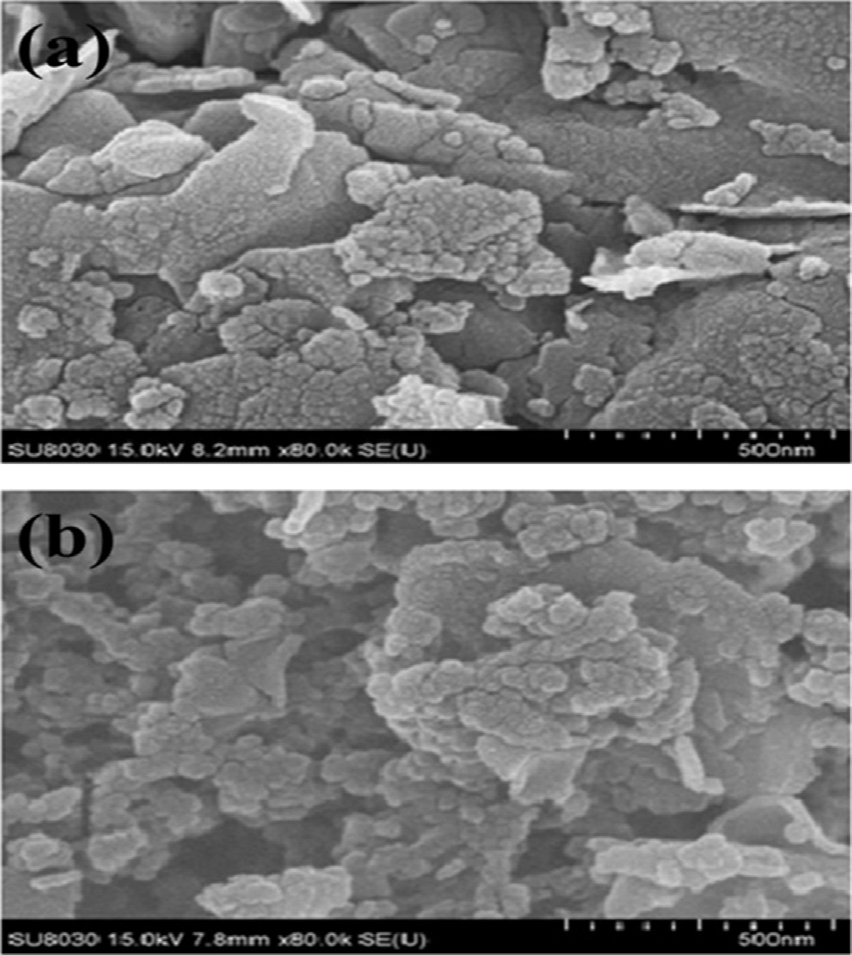
Surface morphological properties of (a) bentonite and (b) modified clay.

Fig. 2 exhibits the morphological properties of modified clay (Fig. 2b) and bentonite (Fig. 2a). The magnification of 80,000× was employed for observation. The microstructure of bentonite was significantly changed from plate-like structure to be porous. This was probably due to the reason that removal of cationic surfactant played an important role on extension of intercalation of silica plate, suggesting that chemical modification by using CTAB was successfully

The nitrogen adsorption–desorption isotherm was investigated. The specific surface area was increased 100 m^2^/g. It was superior to bentonite which was only 30 m^2^/g. It was explained that significance on specific surface area can be observed due to removal of water and solvent in calcination process. The pathway of water and solvent was occurred and specific surface area was therefore obtained.

### 1.2 Experimental design, materials and methods

Modified clay was successfully synthesized by surfactant template method. Bentonite was added into 0.1 M of cetyltrimethyl solution at 50 °C and continuously stirred for 5 h. The product was consequently washed and filtered with 500 ml of 1:1% volume of deionized water and methanol in order to remove any impurity. Furthermore, the product was kept in oven at 80 °C for overnight. After that, it was stirred in dodecylamine solution for 5 h and subsequently following by pouring tetraethyl orthosilicate. After that, it was continuously stirred at room temperature for 6 h. The ratio between as-synthesized product, dodecylamine and tetraethyl orthosilicate is 1 g:10 mmol:30 mmol. After reaction, the product was calcined at 700 °C for 3 h. The modified clay was therefore obtained. X-ray diffraction, scanning electron microscope, N_2_ absorption-desorption were employed to identify the structural, morphological properties and specific surface area, respectively.

## References

[1] Kenya M., Meral K., Onganer Y. (2015). Molecular aggregates of Merocyanine 540 in aqueous suspensions containing natural and CTAB-modified bentonite. J. Mol. Struct..

[2] Gurses A. (2010). Monomer and micellar adsorptions of CTAB onto the clay/water interface. Desalination.

